# Survey of Italian pediatricians’ perspectives and knowledge about neonatal screening

**DOI:** 10.1186/s13052-015-0147-1

**Published:** 2015-05-29

**Authors:** Alberto B. Burlina, Giovanni Corsello

**Affiliations:** Division of Inherited Metabolic Diseases, Reference Centre Expanded Newborn Screening Regione Veneto, Department of Pediatrics, University Hospital, Padova, Italy; Pediatrics and Neonatal Intensive Care Units, University of Palermo, Palermo, Italy

**Keywords:** Newborn screening, Expanded newborn screening, Pediatricians, Survey

## Abstract

**Background:**

The goal of newborn screening is early identification of babies with a high risk for disorders that may not be clinically evident at birth, but have severe consequences if untreated. New insight into inherited diseases and the ability to test for numerous diseases using new technique such as tandem mass spectrometry have made it practical to greatly expand the number of conditions tested. The expanded neonatal screening is now available and relatively simple, but this represents only a part of the picture. Positive results require follow-up confirmation. Most disorders screened require confirmatory biochemical or genetic tests and specialist visits. An efficient system is needed for managing the care of affected newborns. Expanded newborn screening is not yet available in all Regions of Italy, but discussions aimed at organizing universal access are underway. If these are successful, the role of the pediatrician as the primary contact with the parents is expected to become even more important.

**Methods:**

We have conducted a survey of Italian pediatricians to assess their familiarity and opinions on newborn screening in general and on expanded newborn screening. All members of the Italian Association of Pediatricians (*n* = 9000) were invited to compile a 10-item questionnaire online.

**Results:**

The response rate was 10 %, corresponding to 605 of 6000 active members. Respondents were from all Regions of Italy, with the highest number of responses coming from Lombardy (138, 22.8 %), Campania and Puglia (n = 61; 10.1 %). Interestingly, expanded neonatal screening was not available in any of these Regions at the time of the survey. Regarding their understanding of neonatal screening in general, most respondents (n = 552; 91.1 %) considered that they had at least a sufficient level of knowledge; however, only 59.6 % thought they had sufficient knowledge of expanded newborn screening.

**Conclusions:**

Successful implementation of a universal expanded NBS program will require efficient procedures for follow-up, diagnosis and treatment to prevent morbidity and mortality of infants and to reduce the period of uncertainty for unaffected families. Pediatricians may need additional training to allow them to fulfill their tasks of coordinating this process while keeping families informed and reassured.

**Electronic supplementary material:**

The online version of this article (doi:10.1186/s13052-015-0147-1) contains supplementary material, which is available to authorized users.

## Background

The goal of newborn screening (NBS) is early identification of babies with a high risk for disorders that may not be clinically evident at birth, but have severe consequences if undiagnosed and untreated. The prototypical condition is phenylketonuria [1], which is initially asymptomatic, but leads to severe irreversible mental impairment if not treated promptly. Alongside vaccination programs, NBS programs represent one of the great public health success stories, improving outcomes and reducing the burden to patients, families and society. In Italy, neonatal screening for phenylketonuria, congenital hypothyroidism, and cystic fibrosis has been mandatory by law since 1992.

New insight into inherited diseases combined with the introduction of tandem mass spectrometry (MS-MS) and the ability to test for numerous diseases using a fast and reliable analysis has led to expansion of the number of conditions tested. Many of the diseases amenable to screening are rare inborn errors of metabolism resulting from a genetic defect that affects an enzyme active in one or more metabolic pathways. These defects cause pathological accumulation of substrates or deficiencies of essential products. Many are involved in the metabolism of fatty acids, organic acids or amino acids/urea cycle [2]. The majority of these disorders present clinical symptoms in the neonatal period and the conditions cause progressive irreversible damage.

Criteria have been established to select disorders for inclusion (Additional file 1) [3, 4]. Factors to be taken into consideration are *disease* morbidity, mortality, natural history and epidemiology; *test* reliability, precision, clinical validity, cost, and the existence of confirmatory tests; *treatment* availability, effectiveness and the availability of medical expertise. Continuous improvements in treatment, in particular the introduction of enzyme substitution therapies and the prospect of gene therapy may further increase the number of diseases included in screening panels. Discussion is currently ongoing on whether to include lysosomal storage diseases in routine screening [5, 6].

At the international level, there is a high level of heterogeneity regarding the decision to conduct expanded NBS and the composition of screening panels [7–10], and there are also examples of considerable regional heterogeneity within countries. In Italy, newborn screening programs are managed at the regional level. Italian Law 104/1992, DPCM of July 9, 1999 mandates newborn screening for phenylketonuria, congenital hypothyroidism and cystic fibrosis, but it also allows the possibility for individual regions to organize screening programs for additional congenital diseases, and screening programs for endocrinopathies and inborn errors of metabolism are performed in some Italian Regions.

Currently, there is substantial dishomogeneity among Italian Regions regarding the implementation of screening programs. Several Regions have expanded screening programs in place that cover 20 to 40 rare diseases (Tuscany, Umbria, Liguria, Sardinia, Emilia Romagna, Sicily, Veneto), while some other have experimental programs ongoing (Lazio). In all, about one third of newborns currently undergo expanded screening in Italy. Discussions and planning are underway to introduce it uniformly in all regions. There is need for shared criteria for selecting diseases to screen for and an analysis of possible synergies for the most efficient management of this task and the steps that must follow when a positive screening result is obtained. The screening itself is part of a program in which blood spot analysis represents only the initial part of the picture. To insure adequate analytical sensitivity, there is a consistent number of false positive results that require follow-up confirmation. Most of the disorders screened require confirmatory biochemical or genetic tests and specialist visits. When affected newborns are identified, there must be an efficient system for managing their condition. With the introduction of expanded NBS, there will be more positive results (both true and false) and the role of the pediatrician as the primary contact with the parents is expected to become even more important.

Recommendations on expanded screening from the SISMME and SISN [11] indicate that primary care pediatricians should be familiar with the screening procedure and with diseases that are included in the panel. They should also maintain contacts with the local screening facility and the appropriate specialist care providers, and interact directly with the affected families to keep them informed. Pediatric specialists, on the other hand, should organize the follow-up of patients with positive screening results and share the results with the primary pediatrician. They should also coordinate diagnostic and therapeutic efforts of other specialists, and set up the long-term treatment strategy with the primary pediatrician and the family. It is clear that pediatricians have an important role in the process that will grow if universal coverage is established.

We report the results of a survey conducted among pediatricians from all regions of Italy regarding their knowledge and impression of the concept of NBS in pediatrics, focusing on diagnosis, treatment and healthcare resource management.

## Methods

An invitation to participate in the web-based survey was sent to all 9000 members of the Italian Pediatrics Society (SIP). The survey questionnaire was developed by the Authors and consisted of ten questions to gauge familiarity with the principles of NBS and how they apply to the pediatrician’s Region (see below). Questions were included to confirm also the understanding of criteria for selecting diseases to include in the extended panel and the principle characteristics of extended screening.

### Questionnaire on the concept of neonatal screening in pediatrics

1. How would you define your knowledge regarding neonatal screening? (poor, insufficient, sufficient, appropriate)
2. Is neonatal screening performed in your Region? (yes, no, I don’t know)
3. Indicate which region you live in. (Choose from list)
4. Which conditions are screened in your Region? (phenylketonuria and hypothyroidism; cystic fibrosis, galactosaemia, biotinidase deficiency, maple syrup urine disease, congenital adrenal hyperplasia, I don’t know)
5. How would you define your knowledge of expanded newborn screening for inherited metabolic diseases? (poor, insufficient, sufficient, appropriate)
6. Is expanded neonatal screening performed in your Region? (yes, no, I don’t know)
7. Do you think that expanded newborn screening should be required by law? (yes, no, I don’t know)
8. Indicate up to three criteria that a disease should have to be eligible for early detection in a screening program.
  It must be possible to diagnose the disease in the initial phase.
  These are primarily common diseases that are preventable.
  Treatment in the early phase must be more efficacious than that in later phases.
  The possibility of performing the test at any time during preschool age.
  The test should be readable in any Analytical Laboratory.
  The costs must be balanced by the benefits.
9. Indicate two of the main characteristics of expanded neonatal screening:
  Discover the presence of the disease at the first emergence of symptoms
  Diagnose a disease also in the absence of evident symptoms
  The test uses methods that are complicated and expensive
  The test must be performed during the first days after birth
  The test must be performed in the first year after birth
10. Are there reference Centers for treating metabolic disorders in your Region? (yes, no, I don’t know)

## Results and discussion

Surveys were compiled by 605 pediatricians representing all Regions of Italy. The response rate was 10 % of 6000 active members. Detailed results are available in the supplemental material (http://surveys.biomedia.net/screeningchart.html). Respondents were from all Regions of Italy, with the largest number, about one fifth, residing in Lombardy (*n* = 138, 22.8 %); Valle d’Aosta registered a single respondent. Many respondents (44.1 %) reported living in Regions where expanded screening is carried out; however, this number may include regions with partial coverage or where experimental programs are in place.

### Newborn screening

Most respondents (99 %) indicated correctly that neonatal screening is performed in their Region. In fact, basic NBS is mandatory in Italy. Regarding their understanding of neonatal screening in general, most respondents considered that they had at least a sufficient level of knowledge (n = 552, 91.1 %). Regarding the criteria for including a disease in a mass screening panel, responses were consistent with the criteria introduced by Wilson and Jungner [11] and subsequently refined [12]. The most common responses were “diagnosable in the initial phase” (21.5 %), “treatment must be more efficacious when administered early” (30.9 %), “the cost of treating the disease in adults could be unsustainable for the healthcare system” (21.5 %).

### Expanded newborn screening

Nearly 80 % of respondents thought that *expanded* NBS should be required by law. This would appear to reflect a strong belief in the benefit of this service. Responses to the question regarding the principle characteristics of expanded neonatal screening were consistent with a clear understanding of the subject. The two correct answers received a total of 88 % of the selections; however, 40.4 % of respondents felt that their knowledge of *expanded* NBS was insufficient or poor. Interestingly, many of these respondents may live in Regions that do not yet provide expanded screening; nonetheless, this indicates that there is a need for training and education so that these pediatricians will be prepared to inform parents in the event that a harmonized nationwide program is organized to offer screening to all newborns in Italy.

### Follow-up

Finally, the question regarding the presence of reference Centers for metabolic diseases in the respondent’s Region revealed that 537 (89.4 %) believed that there was such a centre in their Region. To some extent, this may reflect more on the percentage of respondents from Regions that have such Centers, but it could also reflect confusion over the definition of a specialist referral centre. Expanded NBS covers diseases that require highly trained metabolic pediatricians or geneticists. Moreover, the Center should have an intensive care unit and pediatricians/neonatologists should be aware that neonatal onset metabolic diseases like urea cycle disorders or organic acidemias may present clinically before screening results are available.

## Conclusions

Primary care pediatricians in Italy are now familiar with NBS using the traditional panel, but some may not be fully aware of the diseases included in *expanded* NBS panels. A large majority of respondents believe that *expanded* NBS should be required by law in Italy. This is an important message from the survey. We need to work toward a uniform screening panel to avoid Regional disparities that might limit the benefit of the screening itself. Clearly, this would entail a substantial increase in the number of callbacks and a corresponding increase in interactions between pediatricians, specialists, laboratories and the families of affected newborns. The information exchanged would be more complex, involving lesser-known diseases. Some of these new diseases do not follow the clear pattern seen in phenylketonuria, where a complete absence of symptoms in the early months is followed by serious neuropsychological damage. Some conditions may even remain asymptomatic “mild cases”, complicating the interpretation of results. Two in five survey respondents did not believe that they had sufficient knowledge about *expanded* NBS. Successful implementation of an expanded NBS program will require resources and efficient procedures for follow-up, diagnosis and treatment to prevent morbidity and mortality of infants and to reduce the period of uncertainty for unaffected families. Pediatricians may need additional training to allow them to fulfill the tasks of coordinating this process while keeping families informed and reassured. The Italian Pediatrics Society can play an important role. Collecting and analyzing results from all Centers will allow benchmarking and future optimization of the screening program in our Country.

## Additional file

Additional file 1: **Scoring systems according to ACMG (2006) and SIMMESN for selecting metabolic diseases to screen.** ACMG score: score reported in the 2006 ACMG report calculated by averaging the scores of at least two experts in the field who have rated the evidence on a scale of 1 to 4 in favor of or against the inclusion of a given condition in the panel, considering the four elements (disease, screening test, diagnosis, and treatment). SIMMESN criteria. Strength of recommendation: A) Strong recommendation for adoption; B) Recommendation of adoption; C) Recommendation to not adopt; I) Evidence insufficient to make a recommendation. Grade of evidence: 1) From well-designed and well-conducted studies on representative populations, 2) Sufficient to determine the effects, but limited by the number, quality or consistency of the individual studies, by their generalizability to routine practice, or by the indirect nature of the evidence, 3) Insufficient to determine effects due to limitations in the number and value of the studies, to imperfections in the design or conduct, to gaps in the chain of evidence 4) Eligible for further pilot studies.

## Abbreviation

NBS: Newborn screening
