# Supplementary material for: Survey of Italian pediatricians’ perspectives and knowledge about neonatal screening
Source: Ital J Pediatr. 2015 May 29;41:41. doi: 10.1186/s13052-015-0147-1 (PMC4462014; doi:10.1186/s13052-015-0147-1)
Supplement: Supplementary file 1 — Scoring systems according to ACMG (2006) and SIMMESN for selecting metabolic diseases to screen. ACMG score: score reported in the 2006 ACMG report calculated by averaging the scores of at least two experts in the field who have rated the evidence on a scale of 1 to 4 in favor of or against the inclusion of a given condition in the panel, considering the four elements (disease, screening test, diagnosis, and treatment). SIMMESN criteria. Strength of recommendation: A) Strong recommendation for adoption; B) Recommendation of adoption; C) Recommendation to not adopt; I) Evidence insufficient to make a recommendation. Grade of evidence: 1) From well-designed and well-conducted studies on representative populations, 2) Sufficient to determine the effects, but limited by the number, quality or consistency of the individual studies, by their generalizability to routine practice, or by the indirect nature of the evidence, 3) Insufficient to determine effects due to limitations in the number and value of the studies, to imperfections in the design or conduct, to gaps in the chain of evidence 4) Eligible for further pilot studies. [file 13052_2015_147_MOESM1_ESM.docx]

***Additional file 1. Scoring systems according to ACMG (2006) and SIMMESN for selecting metabolic diseases to screen***

| **GROUP** | **DISEASE** | **ACMG SCORE 2006** | ***SIMMESN*  CRITERIA** |
| --- | --- | --- | --- |
| AA | Phenylketonuria | 2.00 | A 1 |
| AA | Benign hyperphenylalaninemia | n/a | A 3 |
| AA | Deficit in tetrahydrobiopterin biosynthesis | 2.00 | A 2 |
| AA | Deficit in tetrahydrobiopterin regeneration | 2.50 | A 2 |
| FAO | Medium-chain acyl-CoA dehydrogenase deficiency (MCAD) | 1.63 | A 1 |
| OA | Glutaric acidemia Type I | 2.25 | A 2 |
| OA | Isovaleric acidemia | 1.33 | A 2 |
| AA | Maple syrup urine disease | 2.13 | A 2 |
| AA | Tyrosinemia type I | 1.94 | A 2 |
| FAO | Carnitine uptake deficiency | 2.25 | A 2 |
| FAO | Long-chain 3-hydroxyacyl-CoA dehydrogenase | 2.75 | A 2 |
| FAO | Trifunctional protein deficiency | 3.50 | A 2 |
| FAO | Very long-chain acyl-CoA dehydrogenase | 2.58 | A 2 |
| OA | 3-hydroxy-3-methylglutaryl-CoA lyase deficiency | 2.13 | A 2 |
| OA | Beta ketothiolase deficiency | 3.50 | A 2 |
| OA | Methylmalonic acidemia (Cbl A,B) | 2.75 | A 2 |
| OA | Methylmalonic acidemia (Mut) | 2.57 | A 2 |
| OA | Propionic acidemia | 1.50 | A 2 |
| OA | Methylmalonic acidemia (Cbl C,D) | 2.75 | B 2 |
| AA | Argininosuccinic acidemia | 2.50 | A 2 |
| AA | Citrullinemia | 3.00 | A 2 |
| AA | Homocystinuria disease (CBS deficiency) | 2.00 | A 2 |
| AA | Argininemia | 3.50 | B 4 |
| AA | Citrullinemia type II | 2.71 | B 2 |
| AA | Hypermethioninemia | 1.75 | B 4 |
| AA | Tyrosinemia type II | 2.38 | A 2 |
| AA | Tyrosinemia type III | 3.63 | B 2 |
| FAO | Carnitine palmitoyltransferase I (CPT I) deficiency | 3.75 | B 2 |
| FAO | Carnitine palmitoyltransferase II (CPT II) deficiency | 3.38 | A 2 |
| FAO | Glutaric acidemia Type II | 3.38 | A 2 |
| FAO | Short chain acyl-CoA dehydrogenase deficiency | 2.63 | B 2 |
| FAO | Carnitine-acylcarnitine translocase (CACT) deficiency | 2.58 | B 2 |
| OA | 3-methylcrotonyl-CoA carboxylase deficiency | 2.63 | B 2 |
| OA | Multiple carboxylase deficiency | 2.33 | A2 |
| OA | 2-Methylbutyryl-CoA dehydrogenase deficiency | 2.00 | B 2 |
| OA | 3-Methylglutaconic aciduria | 2.50 | B 2 |
| OA | Isobutyryl-CoA dehydrogenase deficiency | 2.13 | B 2 |
| OA | Malonic aciduria | 4.00 | B 2 |
| AA | Nonketotic hyperglycinemia | n/a | I |
| AA | Pyruvate carboxylase (PC) deficiency | n/a | I |
| FAO | 2,4-dienoyl-CoA reductase deficiency | 4.00 | I |
| FAO | Short-chain-3-hydroxyacyl-CoA dehydrogenase (SCHAD) deficiency | 4.00 | I |
| FAO | Medium-chain ketoacyl-CoA thiolase deficiency (MCAT) | 4.00 | I |
| OA | 2-methyl-3-hydroxybutyric acidemia | 3.75 | I |
| OA | Ethylmalonic encephalopathy | n/a | I |

ACMG score: score reported in the 2006 ACMG report calculated by averaging the scores of at least two experts in the field who have rated the evidence on a scale of 1 to 4 in favor of or against the inclusion of a given condition in the panel, considering the four elements (disease, test, diagnosis, and treatment).

SIMMESN criteria. Strength of recommendation: A) Strong recommendation for adoption; B) Recommendation of adoption; C) Recommendation to not adopt; I) Evidence insufficient to make a recommendation. Grade of evidence: 1) From well-designed and well-conducted studies on representative populations, 2) Sufficient to determine the effects, but limited by the number, quality or consistency of the individual studies, by their generalizability to routine practice, or by the indirect nature of the evidence, 3) Insufficient to determine effects due to limitations in the number and value of the studies, to imperfections in the design or conduct, to gaps in the chain of evidence 4) Eligible for further pilot studies.
